# The Local Topological Free Energy of the SARS-CoV-2 Spike Protein

**DOI:** 10.3390/polym14153014

**Published:** 2022-07-26

**Authors:** Quenisha Baldwin, Bobby Sumpter, Eleni Panagiotou

**Affiliations:** 1 Department of Biology, Tuskegee University, Tuskegee, AL 36088, USA; qbaldwin8382@tuskegee.edu; 2 Center for Nanophase Materials Sciences, Oak Ridge National Laboratory, Oak Ridge, TN 37831, USA; sumpterbg@ornl.gov; 3 Department of Mathematics and SimCenter, University of Tennessee at Chattanooga, Chattanooga, TN 37403, USA

**Keywords:** SARS-CoV-2, Spike protein, topology, mutations, writhe

## Abstract

The novel coronavirus SARS-CoV-2 infects human cells using a mechanism that involves binding and structural rearrangement of its Spike protein. Understanding protein rearrangement and identifying specific amino acids where mutations affect protein rearrangement has attracted much attention for drug development. In this manuscript, we use a mathematical method to characterize the local topology/geometry of the SARS-CoV-2 Spike protein backbone. Our results show that local conformational changes in the FP, HR1, and CH domains are associated with global conformational changes in the RBD domain. The SARS-CoV-2 variants analyzed in this manuscript (alpha, beta, gamma, delta Mink, G614, N501) show differences in the local conformations of the FP, HR1, and CH domains as well. Finally, most mutations of concern are either in or in the vicinity of high local topological free energy conformations, suggesting that high local topological free energy conformations could be targets for mutations with significant impact of protein function. Namely, the residues 484, 570, 614, 796, and 969, which are present in variants of concern and are targeted as important in protein function, are predicted as such from our model.

## 1. Introduction

The severe acute respiratory syndrome coronavirus 2 (SARS-CoV-2) led to the COVID-19 global pandemic that has taken over 6 million lives. Stopping the spread of the highly infectious virus requires disruption of the infection process and has become the focus for many scientists. Furthermore, the ability to control and stop a global pandemic in the future is of great interest. Therefore, understanding the mechanisms of viral infection is a pressing problem. Fusion of the membranes of both a host cell and a viral cell is necessary for infection [1]. Viral glycoproteins aid in this process by facilitating the binding of the two cells. Viral glycoproteins are folded proteins on the enveloped viral cell membrane, which, when triggered, undergo dramatic irreversible conformational changes [2,3,4,5,6,7,8]. Understanding the conformational changes of viral proteins is a challenge due to the multiple length scales involved (e.g., that of consecutive bonds, that of secondary structure elements, or that of tertiary structure of a protein). In this paper, we use tools from topology alone and a training set of more than 13,000 structures from the Protein Data Bank [9] to rigorously characterize the 3-dimensional conformations of the SARS-CoV-2 Spike protein.

Folded proteins are defined by their primary, secondary, and tertiary structure [10]. The secondary structure refers to a sequence of 3-dimensional building blocks the protein attains (beta sheets, α-helices, coils). The tertiary structure refers to the 3-dimensional conformation of the entire polypeptide chain. The rearrangement of viral proteins during protein fusion changes both their secondary and their tertiary structure. Rigorous methods to characterize these changes are necessary. In recent decades, measures from knot theory have been applied to biopolymers [11,12,13,14,15,16,17,18,19,20,21,22,23,24,25,26,27,28,29,30] and in particular to proteins to classify their conformations [27,28,29,30,31,32,33,34,35,36]. One of the simplest measures of conformational complexity of proteins dates back to Gauss: the writhe of a curve [37,38,39,40,41,42,43]. The writhe and the torsion can be used to study the conformation of a protein at different length scales. In [44], the writhe and the torsion were used to define a novel topological/geometrical free energy that can be assigned locally to a protein at the length scale of four amino acids. The results therein showed that high local topological free energy conformations are independent of the local sequence and may be involved in the rate limiting step in protein folding.

In this paper, using a training set of more than 13,000 protein structures as in [44], we apply this method to the Spike protein of SARS-CoV-2 to characterize its conformation in various phases of viral fusion. The SARS-CoV-2 Spike protein is a class I viral glyco-protein that consists of two subdomains (S1 and S2) and is triggered by cleavage at the S1 cleavage site [2,6,45,46,47,48,48]. The receptor binding domain (RBD), which is part of the S1 domain, binds to a host cell receptor, angiotensin-converting enzyme 2 (ACE2), and leads to a second cleavage at an S2’ cleavage site, adjacent to the fusion peptide (FP). The protein undergoes several structural rearrangements, aided by the fusion peptide, which is part of the S2 domain, that lead to a post-fusion state that brings the two membranes together [2]. In this manuscript, we use the local topological free energy to understand how the local geometry/topology of the proteins changes as it undergoes global conformational changes and the effect of mutations on the SARS-CoV-2 Spike protein local and global conformation. Our results on the Spike protein of SARS-CoV-2 show that the known large conformational changes of the RBD are coupled with local conformational changes in the FP, HR2, and CH domains, which are part of the S2 domain. All the analyzed variants in this manuscript (the alpha, beta, gamma, delta, G614, Mink, and N501) show local conformational changes among one another in the S2 domain as well. Our results suggest that higher local topological free energy in these regions is associated with experimentally reported higher propensity of RBD-up conformations and facilitation of fusion [49,50,51,52]. In addition, we find that most important mutations of concern (defined by the WHO, https://www.who.int/en/activities/tracking-SARS-CoV-2-variants/, accessed on 1 January 2022) are in or in the vicinity of high local topological free energy conformations. These results could be very helpful not only in understanding the mechanisms of protein rearrangement using rigorous mathematical tools, but also for predicting mutations with impact on the 3-dimensional structure of proteins.

## 2. Materials and Methods

In this section, we give the definitions of measures of conformational complexity and the local topological free energy of proteins.

### 2.1. Measures of Topological/Geometrical Complexity

We represent proteins by their α carbon (CA) atoms as linear polygonal curves in space. This type of coarse-grained representation of a protein is common when analyzing their structure with topological metrics as well as when analyzing the sequence-distant contacts of a protein [31,53,54]. A measure of conformational complexity of curves in 3-space is the Gauss linking integral. When applied to one curve, this integral is called the writhe of a curve:

**Definition** **1.** *(Writhe). For an oriented curve ℓ with arc-length parameterization γ(t), the writhe, Wr, is the double integral over l:*

(1) $$Wr\left(l\right)=\frac{1}{4\pi }\int_{{\left[0,1\right]}^{*}}\int_{[0,1]\&*}\frac{\left(\gamma^{\prime }\left(t\right),\gamma^{\prime }\left(s\right),\gamma \left(t\right)-\gamma \left(s\right)\right)}{||\gamma \left(t\right)-{\gamma \left(s\right)||}^{3}}dtds$$

where ${\left[0,1\right]}^{*}\times {\left[0,1\right]}^{*}=\left\{s,t\in \left[0,1\right]\times \left[0,1\right]|s\neq t\right\}$.

Writhe is a measure of the number of times a chain winds around itself and can have both positive and negative values.

The total torsion of the chain describes how much it deviates from being planar and is defined as:

**Definition** **2.** *The torsion of an oriented curve ℓ with arc-length parameterization γ(t) is the integral over l:*

(2) $$T\left(l\right)=\frac{1}{2\pi }\int_{\left[0,1\right]}\frac{\left(\gamma^{\prime }\left(t\right)\times \gamma^{\prime \prime }\left(t\right)\right)\cdot \gamma^{\prime \prime \prime }\left(t\right)}{\left|\right|\gamma^{\prime }\left(t\right)\times \gamma^{\prime \prime }\left(t\right){\left|\right|}^{2}}dt.$$

Writhe and torsion have been applied successfully to study entanglement in biopolymers and proteins in particular [27,28,29,37,38,39,40,41,42,43,55,56,57,58,59,60,61,62,63].

For a polygonal curve, the writhe has a simpler expression that does not require numerical integration, as in [64]. The torsion of a polygonal curve also has a finite form, as explained in [64]. Namely, for a polygonal curve, the torsion is equal to the normalized sum of dihedral angles of the chain.

An important property of the writhe and torsion, which makes them useful in practice, is that they can be applied to polygonal curves of any length to characterize 3-dimensional conformations at different length scales. The writhe and the torsion can be applied to any polygonal curve of four or more vertices. In proteins, the writhe and the torsion have been applied at the length scale of the entire protein backbone in [30,65], where it was shown that the logarithm of the experimental folding rates of 2-state and multi-state proteins correlates with the writhe and the torsion of the native state. The writhe and the torsion can be applied to any protein structure, provided its coordinates in 3-space. Thus, they can be applied to any protein with either an experimentally known structure, or, given structures from simulations, even for disordered proteins. In this work, we use the writhe and the torsion to characterize the local conformation of parts of the protein at the length scale of four amino acids; we call this the *local writhe*, *local torsion*, respectively.

**Definition** **3.** *We define the local writhe (resp. torsion) of an amino acid, represented by the CA atom i to be the writhe (resp. torsion) of the protein backbone connecting the CA atoms i,i+1,i+2,i+3.*

**Figure 1:**
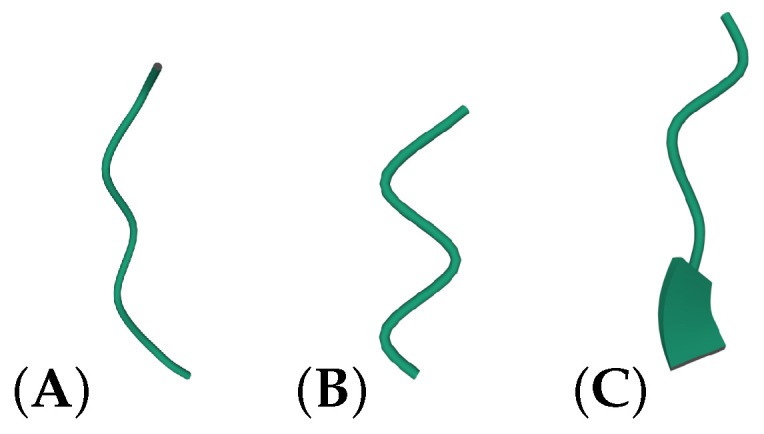
**Examples of local writhe values on the SARS-CoV-2 Spike protein (PDB structure: 6ZGE):** (**A**) residues 9–12: Wr=0.0014, T=0.2504; (**B**) residues 55–58: Wr=0.11, T=0.2499; (**C**) residues 59–62: Wr=−0.00025, T=−0.2507.

The local writhe is a measure of the local orientation of a polygonal curve and a measure of its compactness. For example, a very tight right-handed turn (resp. left-handed) will have a positive (resp. negative) writhe value close to 1 (resp. −1), whereas a relatively straight segment will have a value close to 0. Similarly, the torsion is 0 for a planar segment and increases to ±1 as the segment deviates from being planar. However, such extremes are not observed in proteins. See Figure 1 for some illustrative examples of local conformations in the Spike protein and their corresponding writhe and torsion values.

The writhe and the torsion, as well as the local writhe and local torsion, of proteins can be measured using the software available in https://github.com/TEPPP-software/TEPPP, accessed on 15 July 2022. For a more detailed description of the software, see [66].

### 2.2. Topological/Geometrical Free Energy

In this section, we give the definition of the local topological free energy, originally defined in [44]. To assign a topological/geometrical free energy along a protein backbone, we first derive the distributions of the local writhe and local torsion in the ensemble of folded proteins. To do this in practice, we use a curated subset of of 13,192 proteins from the PDB [9]. Namely, we use the dataset of unbiased, high-quality 3-dimensional structures with less than 60% homology identity from [67], which serves as a representative sample from the PDB, previously also used for similar purposes in [8]. Next, for each amino acid of a given protein, we compare its local writhe (or local torsion) value to those of the ensemble, and a value, which we call local topological free energy, is assigned to the amino acid based on the population of that value in the ensemble.

Namely, let *p* be a value of writhe (resp. torsion) and let d(p) denote the density (i.e., the number of occurrences) of the value *p* in the folded ensemble (the curated ensemble described above). Let *m* denote the maximum occurrence value for writhe (resp. torsion). To any value *p* of writhe (resp. torsion), we associate a purely topological/geometrical free energy:

(3) Π(p)=ln[d(m)/d(p)]

**Figure 2:**
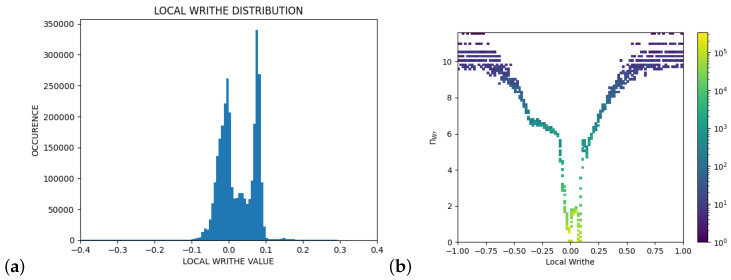
(**a**) The distribution of the local writhe values in the PDB sample. (**b**) The local topological free energy in writhe, $\Pi_{Wr}$, in the PDB sample. Figure from [44]. The distribution of local torsion and $\Pi_{T}$ can be found in [44].

We denote $\Pi_{Wr}$ the Π value in writhe, and $\Pi_{T}$, the Π value in torsion. (See Figure 2 for the distribution of local writhe values and $\Pi_{Wr}$ in the PDB sample.) In the main manuscript, we show the results of the local topological free energy in writhe, whereas the results in torsion are discussed in the Supplementary Information.

We note that the above definition of topological free energy can be applied to different lengths of the protein, by measuring Wr (resp. *T*) for *n* consecutive amino acids at a time. In this manuscript, we will focus on n=4, which is the smallest possible *n* that can be used to define writhe and torsion, and we call it the local topological free energy (LTE). Notice that our sample of more than 13,000 proteins gives rise to more than 1,100,000 local conformations from the PDB. Thus, even if the values of LTE in a protein depend on the original sample used to represent the PDB, we do not expect this choice to influence our model.

The total local topological free energy in writhe (resp. torsion) of a protein is defined as the sum of local topological free energies in writhe along the protein backbone, or along a protein domain. The calculation of the total local topological free energy of a protein follows a sliding window approach, where the local topological free energy of each local conformation of four consecutive CA atoms is added. Similarly, we define the total local topological free energy of a domain of a protein. Notice that in a sliding window approach, there is a partial overlap of conformations and a residue can belong to up to four different conformations, which partially overlap. Even though conformations may overlap, they are not identical and they can have a different LTE.

We will say that a local conformation is *rare* or *in high local topological free energy (high LTE) in writhe (or torsion)* if its value *p* is such that Π(p)≥w, where *w* is a threshold corresponding to the 95th percentile of Π-values across the set of folded proteins. That threshold is $\Pi_{Wr}\geq 2.6$ and $\Pi_{T}\geq 3.5$. For illustrative examples and more information, see [44]. We stress that a high LTE conformation is composed of four consecutive amino acids. We will say that an amino acid is in a high LTE conformation when it is one of these four amino acids composing a conformation with high LTE. In the following, we will refer to such a conformation by the index of its first amino acid.

We note that although the LTE does not have physical units, it has been associated with experimental ϕ values and experimental folding rates of proteins [44]. In addition, note that the total LTE of a domain is affected overall by the secondary structure present in a domain, with helices having smaller normalized total LTE in general. However, the LTE captures more subtle information that does not necessarily correlate with secondary structure. For example, high LTE residues are not found in helices with higher probability than in coils [44]. However, in [44], it was shown that high LTE residues are independent of the amino acid type, which also indicates that atomistic details may not be necessary for analyzing the topological/geometrical structure of the backbone using the LTE. Finally, we stress that the LTE in writhe can be different than that in torsion. For example, high LTE conformations in writhe can be low in torsion and the opposite (see [44] for illustrative examples). We stress that this method can be used to measure the LTE of any protein structure, provided its coordinates are in 3-space. In this manuscript, we focus only on the analysis of structures of the SARS-CoV-2 Spike protein, deposited in the PDB. The aim of this work is to extract rigorous information about the local topology/geometry of the Spike protein in various pre-fusion stages and its variants. We examine how this purely mathematical energy, in the absence of knowledge of the dynamics involved, may point to mechanisms or characteristics of interest related to the protein’s function.

## 3. Results

The SARS-CoV-2 Spike protein is composed of two main domains, the S1 and S2 domains, which are, in turn, composed of other sub-domains. The S1 domain of the protein is composed of the domains NTD, RBD, CTD1, and CTD2. The S2 domain is composed of the domains FP, HR1, CH, CD, and HR2. In between these domains in S2 there are other parts of the protein, which we call A-S2, B-S2, and C-S2.

In the following, we present results on the LTE of SARS-CoV-2 and its domains. In the figures shown in this manuscript, the local topological energy along the protein is shown in the y-axis, and the x-axis represents the backbone of the protein with its domains indicated in the order they are on the protein, namely, NTD, RBD, CTD1, CTD2, A-S2, FP, B-S2, HR1, CH, CD, and C-S2. We present the normalized values of the LTE by the length of the domain. These are the values of the LTE per amino acid in a domain. Note that the Π values range from 0 to approximately 12 (see [44]) and, thus, the normalized total LTE values will be in the same range.

We note that some of the structures analyzed have missing residues. We analyze the LTE of the existing residues in the PDB files only. If there are missing residues in a domain, the LTE of the local conformations existing in the PDB file is normalized by the length of the domain after we subtract the missing residues.

### 3.1. Local Topological Free Energy of SARS-CoV-2 Variants

In this section, we analyze the distribution of the total LTE of the Spike protein of SARS-CoV-2 among its domains for different pre-fusion states and for different variants. We note that the SARS-CoV-2 variants are obtained from different laboratories or different studies. To avoid any inaccuracy, will not aim at comparing the LTE of those structures quantitatively, but rather look at qualitative trends that are apparent in the structures from all studies and across laboratories.

#### 3.1.1. The Distribution of the Total Local Topological Free Energy in the SARS-CoV-2 Spike Protein Domains from Closed to Open Conformation

**Figure 3:**
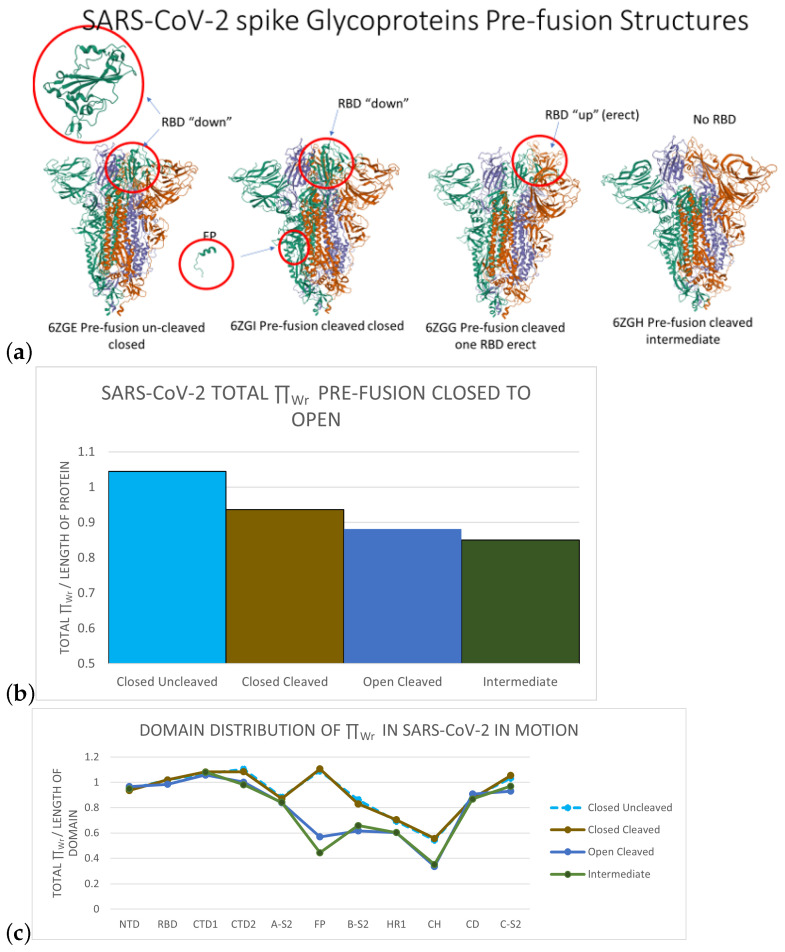
(**a**) From left to right, snapshots of SARS-CoV-2 pre-fusion Spike protein at four stages: uncleaved closed (6ZGE, all 3 RBD down), cleaved closed (6ZGI, all 3 RBD down, proteolytically cleaved), cleaved open (6ZGG, one RBD up), and intermediate (6ZGH, RBD has been removed). (**b**) The normalized total local $\Pi_{Wr}$ for SARS-CoV-2 protein at the four pre-fusion stages. (**c**) The distribution of the normalized total local $\Pi_{Wr}$-values for SARS-CoV-2 protein domains at the four pre-fusion stages. Crystal structure images were pulled from the Protein Data Bank [9].

In pre-fusion, the SARS-CoV-2 glycoprotein may be in uncleaved closed, cleaved closed, cleaved open, and intermediate states. Structures of the protein in each one of these stages were obtained in [2] (see Figure 3 Top). A closed conformation entails that all three RBD are in the down position [2]. A cleaved protein indicates that the protein has been proteolytically cleaved at the cleavage site by a furin protease into the receptor binding subunit of S1, which is necessary for conformational changes of the RBD. An open conformation means that there is an RBD in the up position, accessible for the angiotensin-converting enzyme 2, or ACE2, receptor to bind [2,47,68,69]. An intermediate conformation indicates that cleavage at the RBD has occurred and the RBD has been removed, yet refolding has not occurred [2,69]. In this section, we analyze the local LTE of SARS-CoV-2 in these stages. The structures analyzed in this section were all obtained in similar conditions in [69]. They are all representatives of one of the original forms of the virus, the D614 variant, which we will refer to as the wild-type protein (WT). Because these structures were obtained from the same study with the aim at local comparison of the structure, this enables us also to compare quantitatively the LTE of these structures with confidence.

Figure 3b shows the total LTE of the Spike protein of SARS-CoV-2 in writhe in various stages of rearrangement pre-fusion. Overall, the values are similar in all states, with the largest difference (from closed uncleaved to intermediate) to be 7% of the range of values in the 95th percentile. It is, however, interesting to see a monotonic decrease of the normalized total LTE from the closed cleaved to open and intermediate conformation. We find a similar behavior for the normalized total LTE in torsion (see Supplementary Information). This may suggest a drive towards locally more stable conformations from closed to open state of the WT protein.

Figure 3c shows the distribution of the total LTE in each domain of SARS-CoV-2 normalized by the length of the domain in closed uncleaved, closed cleaved, open, and intermediate conformations. In all states, the B-S2, HR1, and CH domains have lower LTE than the rest of the domains, indicating that they are more stable. This is also in accordance with the mostly helical secondary structure present in these domains. Interestingly, the normalized LTE remains invariant from closed to open state in NTD, RBD, and CTD1 (which compose almost all of S1), whereas it decreases in all the other domains (except A-S2 and CD). This may be surprising, as the RBD domain is the one that is obviously changing position. This suggests that, even if the RBD is changing position relative to the other domains, the local conformations inside the RBD are almost invariant. Instead, our results show that the the biggest small length scale conformational changes in LTE in writhe from closed to open occur in the FP (by 26% of the range of $\Pi_{Wr}$ values in the 95th percentile) and the CH (by 9%) domains of S2 (even though the secondary structures of these domains do not change). This suggests a coupling between local conformational changes in S2 and global conformational changes in S1, an idea that has been supported experimentally [50,52,70]. Similar results are found in torsion in the Supplementary Material.

#### 3.1.2. The Distribution of the Total Local Topological Free Energy in SARS-CoV-2 Protein Variants’ Domains

In this section, we compare the distribution of the total LTE in domains of the WT protein to that of the variants G614, alpha variant, beta variant, gamma variant, delta variant, Mink variant, N501, and HexaPro mutants [71]. The precise impact of these mutations is not yet clear, but current data suggests that the alpha, beta, gamma, and delta mutations may increase both transmission and virulence of the virus. Data suggest that variants increase the ability of a protein to obtain an RBD-up conformation [52]. Not all of the variants have closed or open conformations deposited in the PDB, and for this reason we compare the closed conformations of only WT, G614, alpha, beta, delta, and Mink variants and the open (1 RBD up) conformations of the WT, G614, alpha, beta, gamma, delta, N501, and HexaPro variants. The structures analyzed in this section are obtained from different sources, but the closed conformations come from structures with all the trimer RBD down, and all the open conformations come from structures with only one protein of the trimer with one RBD-up. In order to avoid inaccuracies that may result from the different conditions under which the experimental data were obtained, we will aim at qualitatively comparing the structures and looking for general trends, rather than quantitatively comparing them to derive conclusions.

**Figure 4:**
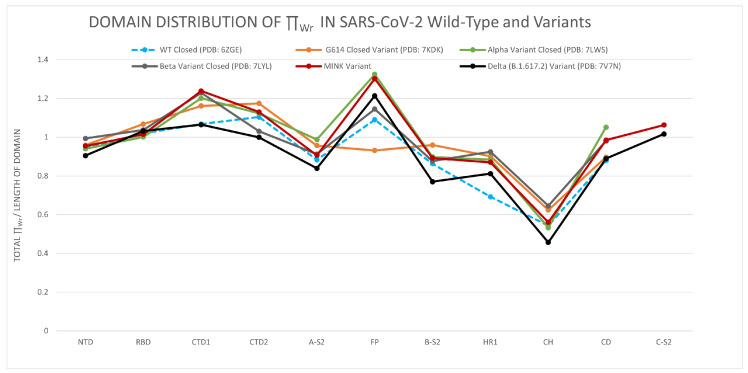
The distribution of the total local topological free energy in the SARS-CoV-2 Spike protein domains for the WT and variants in closed conformations.

In Figure 4, we compare the distribution of the total LTE in the protein domains in the WT and its G614, alpha, beta, delta, and Mink variants in closed conformation. All variants show the lowest LTE values at the BS-2, HR1, and CH domains of S2. Interestingly, the LTE of the RBD is almost invariant among the variants. Our results thus show that mutations affect the local conformations (in writhe) outside the RBD. This is surprising, knowing that mutations affect the propensity of RBD up conformations [50,52,70], but it is in agreement with our results in the previous section. These results thus further corroborate that small length scale conformational changes in S2 have a big impact on the RBD. Similar results are found for torsion in the Supplementary Information. The local torsion captures the local dihedral angle changes. This result thus agrees with what was observed in [70] using time-lagged independent component analysis.

**Figure 5:**
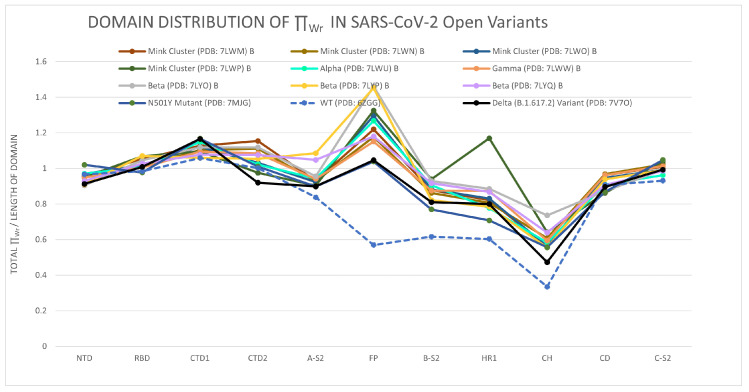
The distribution of the total local topological free energy in SARS-CoV-2 Spike protein domains for the WT and variants in open conformations.

Figure 5 shows the distribution of the total LTE in domains of the alpha, beta, gamma, delta, Mink Cluster, and N501 variants in open conformations. Again, we see that the LTE of the RBD is invariant among variants. Comparing to Figure 4, we also see that all variants show an invariance of total LTE in the RBD from closed to open conformation, in agreement with the results in the previous section. This result again shows that mutations change the local conformations (in writhe) outside the RBD. We find that the largest deviations in the LTE are observed in the FP domain (30% of the range of $\Pi_{Wr}$ values in the 95th percentile). Moreover, the FP, B-S2, HR1, and CH domains have higher LTE for the mutants in open conformation, indicating an instability induced by mutations. Notice that differences in LTE do not necessarily correlate with differences in secondary structure (as was discussed in Section 2.2). In this case, we find secondary structure changes in the variants in FP and HR1, but no secondary structure changes are observed in CH.

The results in torsion show minor local changes in the entire protein (with the maximum at the FP), except in CH, which is invariant for all variants. This suggests a different effect of mutations on the local torsion and local writhe in the open conformations, which is discussed in the Supplementary Information.

### 3.2. High Local Topological Free Energy Conformations in SARS-CoV-2

The high LTE conformations in the SARS-CoV-2 Spike protein at various stages of pre-fusion, post-fusion, and for its variants are given in Table 1 and Table 2. (For the definition of high LTE conformations, see Section 2.2.) The local conformations are denoted by the first amino acid they are composed of. Thus, the table lists only those initial amino acids. In [44], it was shown that high LTE conformations are independent of local sequence. As high LTE conformations are related to the rate limiting step in protein folding [44], these local conformations may be important for the folding of the protein.

We report the high LTE conformations for the G614, the alpha, beta, gamma, delta, Mink, and N501 variants. Although these structures are obtained from different experiments and they can have local differences, we find that some of the high LTE conformations are conserved in all the variants of SARS-CoV-2. More precisely, we notice that amino acids 110, 122, 197, 281, 308–338–365, 480, 543, 569, 601, 612–614, 667, 743, 796, 797, 855, 908–913, 963–964, and 969 are in high LTE conformations in writhe or in torsion in almost all the structures of the Spike protein analyzed in this manuscript. We notice that the majority of those conserved high LTE conformations are outside the RBD.

**Figure 6:**
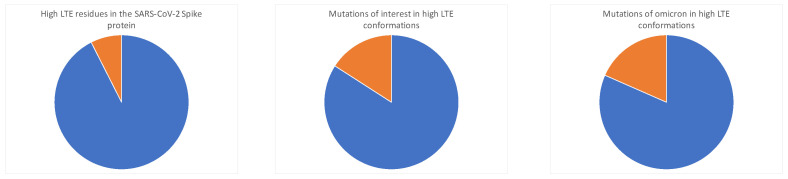
**High LTE conformation proportions.** We find a disproportional number of mutations of interest occur in high LTE conformations. Namely, only 8% of local conformations in SARS-CoV-2 have high LTE. However, 18% of mutations of interest occur in high LTE conformations. Out of all the mutations in the omicron variant, 22% are in a high LTE conformation.

We compare the observed high LTE conformations to the locations of the actual natural mutations of the Spike protein. We find that only 8% of the residues of the Spike protein are in a high LTE conformation, but that 18% of the natural mutations of interest (as defined by the WHO https://www.ecdc.europa.eu/en/covid-19/variants-concern, accessed on 1 January 2022) are in a high LTE conformation; see Figure 6. In addition, we find that all of the dominant lineages (lineages associated with variants that have become prevalent in the population) contain mutations in high LTE conformations (involving mutations at 614, 484, and 570). This suggests that mutations in high LTE conformations have a selective advantage over other mutations. Based on the results of [44], we may hypothesize that mutations that reside in high LTE conformations may have the advantage of not impacting the local topological energy but possibly have larger length scale effects.

We also compare our results to those of engineered mutations. Engineered mutations are obtained from controlled systematic experimental substitutions of amino acids one or multiple at a time, whose observed effect on the protein structure and function was reported in [50,71,72] (data shown in the Supplementary Information). When focusing on engineered mutations, we find that 75% of the engineered mutations that are known experimentally to change the 3-dimensional properties of the Spike protein are at amino acids in high LTE conformations.

Our analysis thus predicts conserved high LTE residues to be advantageous locations for mutations. Indeed, the novel variant of concern, the omicron variant (for which a crystal structure is not deposited in the PDB at the time of writing this manuscript), which became dominant in January 2022, has mutations at two additional conserved high LTE residues compared to the previous variants. Summarizing, thus, out of the conserved high LTE residues, 480, 543, 569, 612, 796, 855, and 969 are related to natural mutation lineages that involve the mutations 614, 570, and 484, whereas 796 and 969 are related to mutations in the omicron variant. Therefore, we find that even though most natural mutations do not occur at high LTE residues, high LTE conformations have higher probability of containing a natural mutation and that most conserved high LTE residues are associated with mutations (see Figure 6). In addition, most engineered mutations known to affect 3-dimensional conformation are associated with high LTE residues. These results point to high LTE residues as locations where mutations affect the 3-dimensional structure of the protein.

## 4. Discussion

We used a novel method to analyze the local structure of proteins to detect subtle local differences in the structure of SARS-CoV-2 in various pre-fusion stages and across different variants. More precisely, using a training set of more than 13,000 proteins, we obtained a parameter associated with 3-dimensional protein structure, the local topological free energy, which enables a novel characterization of proteins. We apply this method to characterize the SARS-CoV-2 Spike protein backbone. We find that the total local topological free energy of the Spike protein pre-fusion decreases in the steps leading to protein fusion. The distribution of the local topological free energy in the domains of the Spike protein changes from closed to open states. Interestingly, most changes are observed in the sub-domains of the S2 domain (in particular, the FP, HR1, and CH domains) and not in the RBD domain of S1, which is known to undergo a major global rearrangement. This reveals a coupling between local conformational changes in S2 and major global rearrangement in S1.

All variants analyzed in this manuscript (alpha, beta, gamma, delta, N501, G614, and Mink) show that the total LTE in writhe is invariant in RBD, and most differences are instead found in the FP, HR1, and CH domains of S2. This shows that all the mutations of concern have higher impact on the local conformation of S2 and not that of the RBD, even though mutations are known to impact the propensity of RBD-up conformations. This further corroborates that local conformational changes in S2 can have a big impact on the global 3-dimensional conformation of the protein.

The local topological free energy also enables us to identify locations on the protein backbone where high LTE conformations occur. These are conformations with writhe or torsion outside the 95th percentile of local conformations in the PDB. Our results show that, even though most natural mutations do not occur in high LTE conformations, mutations of concern occur disproportionally to high LTE conformations, compared to the frequency of high LTE conformations in the SARS-CoV-2 backbone. In addition, the most important mutations (associated with variants of concern) occur in or in the vicinity of a high LTE conformation. Thus, high LTE conformations provide an evolutionary advantage for mutations. Compared to experimental data of engineered mutations, we find that most mutations reported to alter the global protein conformation are at amino acids in high LTE conformations. High LTE conformations are conserved overall among variants, suggesting that mutations at these locations do not impact the local structure. This further corroborates that mutations at high LTE conformations provide stability in the local structure of the protein, yet have significant effects on its global conformation. Our results thus point to high LTE amino acids as targets of advantageous mutations with impact on protein folding and function.

These results not only help us understand the mechanisms of viral function, but also predict the effect of mutations at specific locations along the protein. For example, the recently discovered omicron variant has two new mutations in the S2 domain, at two conserved high LTE residues. Our results could be used by experimentalists to examine how mutations at the proposed locations that were identified using mathematics alone may impact viral function. Further applications of this topological analysis to viral proteins could lead to our better understanding and controlling viral protein function.

## Figures and Tables

**Table 1:** High local topological free energy conformations of SARS-CoV-2 (WT).

Spike Glycoprotein pre-fusion (6VSB), Closed chain A
Writhe 95–100% 86, 121, 197, 281, 296, 542, 569, 687, 688, 749, 753, 796, 797, 908, 969,
984, 1083, 1097
Torsion 95–100% 107, 543, 741, 756, 884
**Spike Glycoprotein post-fusion (6XRA, chain A)**
Writhe 95–100% 754, 917, 965, 1083, 1124, 1188, 1192, 1193
Torsion 95–100% 743, 1039, 1090, 1153
**Spike Glycoprotein pre-fusion (6VYB, Open Chain B)**
Writhe 95–100% 281, 302, 337, 569, 601, 667, 796, 797, 855, 908, 942, 969
Torsion 95–100% 31, 86, 122, 462, 543, 612, 743, 756, 884
**Closed Spike Glycoprotein pre-fusion (6VXX, Open Chain B)**
Writhe 95–100% 97, 110, 197, 281, 302, 543, 569, 601, 667, 796, 797, 855, 908, 969
Torsion 95–100% 31, 86, 122, 612, 743, 756, 884
**Uncleavable Closed Spike Glycoprotein pre-fusion (6ZGE, chain A)**
Writhe 95–100% 31, 68, 110, 197, 281, 365, 480, 543, 569, 645, 667, 796, 797, 822, 855,
905, 908, 969
Torsion 95–100% 107, 743, 821, 822, 823
**Cleaved Closed Spike Glycoprotein pre-fusion (6ZGI, chain A)**
Writhe 95–100% 31, 110, 197, 281, 365, 480, 543, 569, 667, 796, 797, 822, 855, 908, 969,
1018, 1083, 1097
Torsion 95–100% 107, 743, 821, 822, 823, 1017, 1018, 1019, 1039, 1056, 1082, 1090
**One RBD Erect(open) Spike Glycoprotein pre-fusion (6ZGG, chain B)**
Writhe 95–100% 110, 197, 281, 543, 569, 667, 796, 855, 908, 969
Torsion 95–100% 480, 884
**Cleaved Intermediate Spike Glycoprotein pre-fusion (6ZGH, chain A)**
Writhe 95–100% 97, 110, 197, 281, 543, 569, 796, 855, 908, 985
Torsion 95–100% 107, 741, 969

High LTE conformations in SARS-CoV-2, denoted by the index of their initial amino acid. These are conformations of four amino acids that correspond to the complement of the 95–100th percentiles of the writhe and torsion distributions of a PDB culled ensemble.

**Table 2:** High local topological free energy conformations of SARS-CoV-2 variants.

Spike Glycoprotein pre-fusion G614 Variant, Minus RBD (6XS6, chain A)
Writhe 95–100% 110, 121, 197, 281, 559, 561, 562, 569, 601, 667, 743, 796, 797, 855,
908, 913, 963, 964, 969, 1083, 1097
Torsion 95–100% 107, 196, 612, 1056
**Spike Glycoprotein pre-fusion Closed G614 Variant (7KDK, Chain A)**
Writhe 95–100% 110, 121, 197, 281, 302, 337, 365, 543, 559, 569,
601, 645, 667, 743, 753, 796, 797, 855, 865, 908, 913, 964, 969, 1083, 1097
Torsion 95–100% 107, 612, 1039, 1056
**Open South African (B.1.351) Spike Glycoprotein Variant pre-fusion (7LYL, chain A)**
Writhe 95–100% 97, 110, 121, 136, 197, 281, 302, 338, 368, 480, 525, 543, 568, 569,
601, 667, 743, 753, 796, 797, 855, 908, 913, 964, 969
Torsion 95–100% 31, 212, 462, 612
**Open Brazil (P.1) Spike Glycoprotein Variant pre-fusion, (7M8K, Chain A)**
Writhe 95–100% 67, 110, 197, 281, 543, 569, 601,
612, 667, 743, 753, 796, 797, 855, 908, 969
Torsion 95–100% 122, 462, 485, 611, 613, 884
**Open N501Y Spike Glycoprotein Variant pre-fusion (7MJG, Chain B)**
Writhe 95–100% 97, 197, 281, 480, 543, 569, 601, 667, 796, 797, 908, 969,
988, 1083, 1097
Torsion 95–100% 612, 743
**Open D614G substitution Variant pre-fusion (7KRQ, Chain A)**
Writhe 95–100% 97, 110, 146, 148, 197, 281, 406, 543, 569,
601, 623, 667, 753, 796, 797, 830, 855, 908, 964, 969
Torsion 95–100% 841
**Closed UK (B.1.1.7) SARS-CoV-2 S-GSAS-D614G Variant Spike**
**protein pre-fusion (7LWS, Chain A)**
Writhe 95–100% 110, 121, 197, 281, 365, 386, 406, 480, 525, 543, 569, 601, 667, 742,
743, 796, 797, 908, 913, 963, 969, 1097
Torsion 95–100% 96, 462, 485, 568, 612
**Open UK (B.1.1.7) S-GSAS-D614G Variant Spike Glycoprotein (7LWT, Chain A)**
Writhe 95–100% 121, 197, 281, 302, 338, 365, 406,
480, 543, 569, 667, 743, 796, 797, 811, 855, 908, 913, 963, 964, 969
Torsion 95–100% 107, 462
**Open South African (B.1.351) Spike protein Variant S-GSAS-B.1.351 (7LYN, Chain A)**
Writhe 95–100% 121, 197, 281, 302, 337, 365, 480, 543, 559, 569, 601, 667,
743, 796, 797, 855, 908, 913, 963, 964, 969
Torsion 95–100% 462, 485
**Open Brazilian (B.1.1.28) Spike protein Variant, S-GSAS-B.1.1.28, pre-fusion**
**(7LWW, Chain A)**
Writhe 95–100% 121, 138, 139, 197, 281, 365, 406, 480, 543,
559, 569, 589, 601, 645, 667, 743, 752, 796, 797, 811, 855, 908, 913,
963, 964, 969
Torsion 95–100% 462, 485, 612
**Closed Delta Variant pre-fusion (7V7N, Chain A)**
Writhe 95–100% 97, 195, 279, 363
478, 541, 567, 599, 665, 794, 795, 853, 906, 967, 1081, 1095
Torsion 95–100% 96, 610, 1037, 1054, 1088
**Open Delta Variant (open) pre-fusion (7V7O, Chain A)**
Writhe 95–100% 97, 195, 279, 478, 515, 541, 557,
567, 665, 741, 794, 795, 906, 967, 986, 1081, 1095
Torsion 95–100% 610, 1037, 1088 height

High LTE conformations in SARS-CoV-2, denoted by the index of their initial amino acid. These are conformations of four amino acids that correspond to the complement of the 95–100th of the writhe and torsion distributions of a PDB culled ensemble.

## Data Availability

Not applicable.

## Supplementary Materials

The following supporting information can be downloaded at: https://www.mdpi.com/article/10.3390/polym14153014/s1, Table S1: Mutations of SARS-CoV-2 spike protein variants of concern; Table S2: Engineered mutations of SARS-CoV-2 spike protein with experimentally observed impact on protein rearrangement. Figure S1: Above: The normalized local II in Torsion for the SARS-CoV-2 Spike protein at different pre-fusion states: closed, closed cleaved, open and intermediate. Below: The distribution of the (normalized) LTE in domains of the Spike protein in the 4 pre-fusion stages; Figure S2: The normalized local II in Torsion for the SARS-CoV-2 variants. Above: Closed Spike protein (all RBD down). Below: Open Spike protein (one RBD up). References [46,50,52,70,71,72,73,74,75,76,77,78,79,80,81,82] are cited in the supplementary materials.
