# Supplementary material for: The Local Topological Free Energy of the SARS-CoV-2 Spike Protein
Source: Polymers (Basel). 2022 Jul 26;14(15):3014. doi: 10.3390/polym14153014 (PMC9332627; doi:10.3390/polym14153014)
Supplement: Supplementary file 1 [file polymers-14-03014-s001.zip › polymers-1825758-supplementary.pdf]

Supplementary Materials: The Local Topological Free Energy of the SARS-CoV-2 Spike Protein *

Quenisha Baldwin, Bobby Sumpter and Eleni Panagiotou

1. Mutations of interest of SARS-CoV-2

Here we give a more detailed description of the mutations in the variants studied in this manuscript and high LTE residues in these proteins, as well as engineered mutations. The indices of the mutations studied in this manuscript are shown in Tables S1 and S2.

More precisely:

Natural mutations:

alpha variant: The alpha variant (also called VUI 202012/01 and UK variant) is reported to contain several mutations including deletion mutations (at amino acids 69, 70, 144) and substitution mutations (at 501, 570, 614, 681, 716, 982, 1118) [5]. We find high LTE conformations related to mutations 570, 614 and 982. Amino acid 614 is in high LTE in Torsion and amino acid 570 is in high LTE in Writhe. Also, amino acid 570 makes a hydrogen bond with amino acid 856 upon mutation, which is in a high LTE in Writhe conformation. Amino acid 982 forms a hydrogen bond with amino acid 547 which follows the high LTE conformation in Writhe 543-544-545-546.

beta variant: The beta variant (B.1.351 lineage), involves N501Y, E484K, K417N, and D614G [5]. The amino acids 614 and 485 are in high LTE in Torsion and the amino acid 484 follows the high LTE in Writhe conformation 480-481-482-483.

gamma variant: The gamma variant (B.1.1.28/P.1 lineage), emerged from the UK variant in late 2020, with 11 mutations, D614G, K417T, E484K, N501Y, L18F, T20N, P26S, D138Y, R190S, H655Y, and T1027I, which most are located in the NTD or RBD [15]. Of those, 614 is in high LTE in Torsion and 138 is in high LTE in Writhe. Also, the conformation 480-481-482-483, which preceeds 484, is in high LTE in Writhe in many proteins, including the gamma variant.

delta variant: On April 9th 2021, a new variant originating in India, the delta variant, was reported and identified as B.1.617. The strain carries mutations E484Q (similar to E484K in the SA variant) and L452R (same as a mutation found in the epsilon variant) [2] as well as T478K, D614G, P681R. We find amino acid 485 and 614 in high LTE in Torsion and 484 follow the conformation of 480-481-482-483 which is in high LTE in Writhe.

Mink variant: In April of 2020, a mink variant of SARS-Cov-2 was reported in the Netherlands [5]. The variant has been found to have spread from humans to minks and then back to humans. Five mutations in the Spike protein include deletions of H69 and V70, and substitutions of Y453F, I692V, and M1229I. We find none to be in high LTE conformations.

Engineered mutations

Mutations at SARS amino acids K968 and V969 (known as 2P a double proline mutation) caused disruption of conformational changes upon binding [10]. Amino acid 969 is in a high LTE conformation in Writhe. Amino acid 985, which is involved in a stabilizing mutation, is in a high local topological free energy conformation in the WT and in the delta variant [12].

In [8] 43 substitutions where studied. The most efficient was the HexaPro variant (involves 6 mutations), which stabilized the pre-fusion structure and produced high yield [8]. We find amino acids 942 and 986 and 987 out of the six mutations composing HexaPro (F817P, A892P, A899P, A942P, K986P, V987P) to be in a high LTE conformation in WT.

In [6], using a different method, specific sites for mutation that would change global conformation were identified. These were a double cysteine mutant, S383C D985C (RBD to S2 double mutant (rS2d)), a triple mutant, D398L S514L E516L (RBD to NTD (triple

*Work supported by NSF REU 1852042, DMS 1913180, 1925603 and CAREER 2047587.

mutant (rNt)), a double mutant, N866I A570L (subdomain 1 to S2 double mutant (u1S2d)), a quadruple mutant, A570L T572I F855Y N856I (subdomain 1 to S2 quadruple mutant (u1S2q)) and finally, a double cysteine mutant, G669C and T866C, to link S2 to S2 (subdomain 2 to S2 double mutant (u2S2d)). We found that out of these 5 mutants, 4 (all except the rNt mutant) contained amino acids in high LTE conformations in WT. Moreover, one of the most efficient mutations, u1S2q, contained amino acids in which we find all in a high local topological free energy conformation in all variants.

In [9], a combination of mutations were examined. Categorized by location the mutations included N532P, T572I, D614N, D614G of S1, and A942P, T941G, T941P, S943G, A944P, A944G , and A892P, F888 and G880C, S884C and A893C, and K986P and V987P of the C-terminus of HR1 [9]. A892P was reported to increase closed trimers while A942 decreased closed trimers. The double proline mutation (2P) at K986P and V987P stabilized the pre-fusion structure. K986P was reported to have higher ACE2 binding affinity while D614N and T572I reported to have low binding affinity [9]. We find amino acids 572, 614, 942, 943, 944, 884, 986, 987 are in high LTE conformations.

Table S1. Mutations of SARS-CoV-2 spike protein variants of concern.

Mutations (Natural)	Residue in high LTE	Conformation State
D614G [9,11]	614	almost all pre-fusion
T723T [16]	-	-
Y789Y [16]	-	-
D936Y [16]	-	-
L5F [16]	-	-
T1100T [16]	1100	6XS6, 7KDK, 6VSB, 6ZGI, 7MJG, 7LWS
Q677H	-	-
S943T and S943R[16]	943	6VYB
1124 [16]	G1124	6XRA
P715P [16]	-	-
SARS-CoV-2 VUI 202012/01 [4] (501, 570, 614, 681, 716, 982, 1118)	570, 614	all pre-fusion
B.1.351 lineage SA [5,14] (N501Y, E484K, K417N, D614G)	614, 484	see first row and) 6ZGE, 6ZGI, 7LWT
B.1.1.28 (P.1) lineage[15] (D614G, K417T, E484K, N501Y, L18F, T20N, P26S, D138Y,R190S, H655Y, and T1027)	138, 484, 614	almost all pre-fusion
B.1.617 lineage[1] (I402L, I434K, E484Q, S494P, D614G)	614, 484	almost all pre-fusion
B.1.427/429 lineage [3] (S13I, W152C, and L452R)	-	-
B.1.1.529 lineage (omicron) A67V, del69-70, T95I, del142-144, Y145D, del211, L212I, ins214EPE, G339D, S371L, S373P, S375F, K417N, N440K, G446S, S477N, T478K, E484A, Q493R, G496S, Q498R, N501Y, Y505H, T547K, D614G, H655Y, N679K, P681H, N764K, D796Y, N856K, Q954H, N969K, L981F	339, 478, 547, 614, 484, 796, 856, 969	all pre-fusion

Table S2. Engineered mutations of SARS-CoV-2 spike protein with experimentally observed impact on protein rearrangement.

Mutations (Engineered)	Residue in high LTE	Conformation State
A688 [7]	A688	6VSB
D985C [12]	D985	Intermediate 6ZGH, 6VSB
HexaPro [8]	A942 P986 P987	Open 6VYB, 6ZGH, 6VSB
S383C D985C [6]	D985	6ZGH, 6VSB
N866I A570L [6]	A570	All pre-fusion with RBD
A570L T572I F855Y N856I [6]	T572, F855, N856, A570	All pre-fusion excl. 6VSB
G669C T866C [6]	G669	All pre-fusion with RBD
D614N A892 A942P V987P [9]	A942, P987, D614	6VYB, 6ZGH, 6VSB
D614N A892 A942P V987P K986 [9]	A942, P986, P987, D614	6VYB, 6ZGH, 6VSB
T572I D614N [9]	T572 D614	6VYB, Closed 6VXX, 6VSB
S943G [9]	S943	Open 6VYB
A944P and A944G [9]	A944	Open 6VYB
S884C [9]	S884	6VSB, 6ZGG, 6VYB, 6VXX
D398L S514L E516L [6]	-	-
N532P [9]	525	7LYL, 7LWS
T941G and T941P [9]	942	6VYB
F888C [9]	884	6VSB, 6VYB, 6VXX, 6ZGG, 7M8K
G880C [9]	-	-

2. Local topological free energy in Torsion

In this section we present data on the local topological free energy in Torsion of SARS-CoV-2.

Figure S1 (Above) shows the normalized total LTE in Torsion in closed uncleaved, closed cleaved, open and intermediate conformation. Notice that the Π_T values range from 0 to 14 and that the 95th percentile corresponds to values between 0 and 3.5. Thus, the maximum change from closed uncleaved to intermediate is 3% os the 95th percentile. Nevertheless, we see an overall decrease of the LTE from closed to open conformations.

Figure S1 (Below) shows the distribution of the total LTE in Torsion in domains in pre and post-fusion. We see that the LTE of the RBD is almost invariant, while the FP, CH and CD (all parts of the S1 domain), show the largest differences (up to 28%). This agrees with what was observed for the LTE in Writhe, suggesting that the RBD-up/down conformational changes are coupled with small length scale changes in domains far from the RBD. The local torsion captures the local dihedral changes. This result thus agrees with what was observed in [13] using time-lagged independent component analysis.

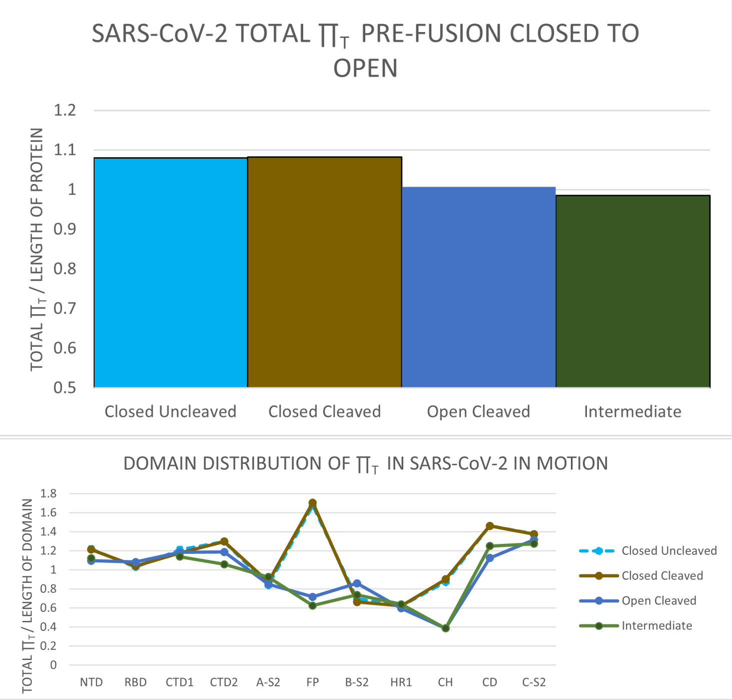

Figure S1. Above: The normalized local Π in Torsion for the SARS-CoV-2 Spike protein at different pre-fusion states: closed, closed cleaved, open and intermediate. Below: The distribution of the (normalized) LTE in domains of the Spike protein in the 4 pre-fusion stages.

Figure S2 (Above) shows the distribution of the normalized total LTE in Torsion in the domains of the Spike protein for different variants in closed conformation (RBD down). The largest changes of the normalized total LTE are observed in the FP and the CH domain between the WT and its variants. This is similar to what was observed for the Writhe. However, the LTE in Torsion shows changes also in the RBD, but the changes observed in the RBD are small relative to the range of values of Π_T (of the order of 5%, compared to the values of Π_T in the 95th percentile). It is however clear that the LTE of the CH of the variants is almost identical. This shows a stability of dihedral angles in the CH, even though their Writhe varies.

Figure S2 (Below) shows the distribution of the normalized total LTE in Torsion in the domains of the Spike protein for different variants in open conformation (RBD up). We see that all the variants have the same LTE in Torsion in the CH domain. The largest difference is still observed in the FP (of the order of 14% relative to the 95th percentile in

Torsion values), but differences are now observed in the RBD as well (of the order of 8%). The LTE in Torsion in the FP is larger for the mutants than that of the WT, suggesting that the FP is less stable in mutants, as was observed for the Writhe.

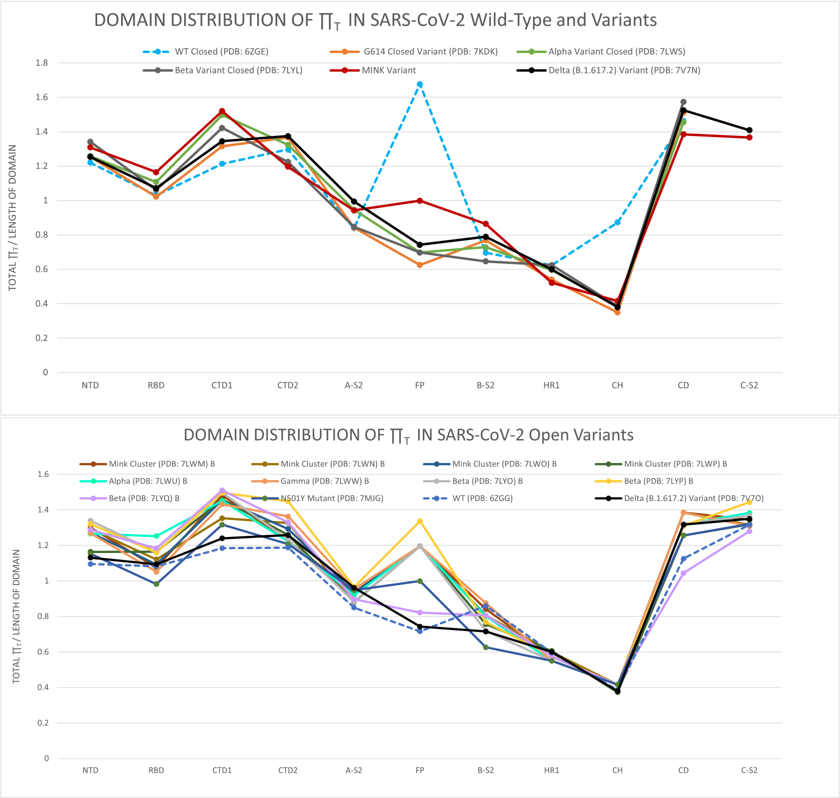

Figure S2. The normalized local Π in Torsion for the SARS-CoV-2 variants. Above: Closed Spike protein (all RBD down). Below: Open Spike protein (one RBD up).

References

1. Alai, S.; Gujar, N.; Joshi, M.; Gautam, M.; Gairola, S. Pan-India novel coronavirus SARS-CoV-2 genomics and global diversity analysis in spike protein. *Heliyon* **2021**, *7*, E06564. [CrossRef] [PubMed]
2. Carroll, T.; Fox, D.; van Doremalen, N.; Ball, E.; Morris, M.K.; Sotomayor-Gonzalez, A.; Servellita, V.; Rustagi, A.; Yinda, C.K.; Fritts, L.; et al. The B.1.427/1.429 (epsilon) SARS-CoV-2 variants are more virulent than ancestral B.1 (614G) in Syrian hamsters. *bioRxiv* **2021**. [CrossRef] [PubMed]
3. Deng, X.; Garcia-Knight, M.A.; Khalid, M.M.; Servellita, V.; Wang, C.; Morris, M.K.; Sotomayor-González, A.; Glasner, D.R.; Reyes, K.R.; Gliwa, A.S.; et al. Transmission, infectivity, and neutralization of a spike L452R SARS-CoV-2 variant. *Cell* **2021**, *184*, 3426–3437.e8. [CrossRef] [PubMed]
4. European Centre for Disease Prevention and Control. *Rapid Increase of a SARS-CoV-2 Variant with Multiple Spike Protein Mutations Observed in the United Kingdom*; European Centre for Disease Prevention and Control: Solna, Sweden, 2020.
5. Gobeil, S.M.C.; Janowska, K.; McDowell, S.; Mansouri, K.; Parks, R.; Manne, K.; Stalls, V.; Kopp, M.; Henderson, R.; Edwards, R.J.; et al. Effect of natural mutations of SARS-CoV-2 on spike structure, conformation and antigenicity. *Science* **2021**, *373*, eabi6226. [CrossRef] [PubMed]
6. Henderson, R.; Edwards, R.J.; Mansouri, K.; Janowska, K.; Stalls, V.; Gobeil, S.M.C.; Kopp, M.; Li, D.; Parks, R.; Hsu, A.L.; et al. Controlling the SARS-CoV-2 spike glycoprotein conformation. *Nat. Strut. Mol. Biol.* **2020**, *27*, 925–933. [CrossRef]
7. Hoffmann, M.; Kleine-Wber, H.; Pohlmann, S. A multibasic cleavage site in the spike protein of SARS-CoV-2 is essential for infection of human lung cells. *Mol. Cell* **2020**, *78*, 779–784.e5. [CrossRef]
8. Hsieh, C.L.; Goldsmith, J.A.; Schaub, J.M.; DiVenere, A.M.; Kuo, H.-C.; Javanmardi, K.; Le, K.C.; Wrapp, D.; Lee, A.G.; Liu, Y.; et al. Structure-based design of prefusion-stabilized SARS-CoV-2 spikes. *Science* **2020**, *369*, 1501–1505. [CrossRef] [PubMed]
9. Juraszek, J.; Rutten, L.; Blokland, S.; Bouchier, P.; Voorzaat, R.; Ritschel, T.; Bakkers, M.J.G.; Renault, L.L.R.; Langedijk, J.P.M. Stabilizing the closed SARS-CoV-2 spike trimer. *Nat. Commun.* **2021**, *12*, 244. [CrossRef] [PubMed]
10. Kirchdoerfer, R.N.; Wang, N.; Pallesen, J.; Wrapp, D.; Turner, H.L.; Cottrell, C.A.; Corbett, K.S.; Graham, B.S.; McLellan, J.S.; Ward, A.B. Stabilized coronavirus spikes are resistant to conformational changes induced by receptor recognition of proteolysis. *Sci. Rep.* **2018**, *8*, 15701. [CrossRef]

11. Will, B.K.; Fischer, M.; Gnanakaran, S.; Yoon, H.; Theiler, J.; Abfalterer, W.; Hengartner, N.; Giorgi, E.E.; Bhattacharya, T.; Foley, B.; et al. Tracking changes in SARS-CoV-2 spike: Evidence that d614g increases infectivity of the COVID-19 virus. *Cell* **2020**, *182*, 812–827.e19.
12. McCallum, M.; Walls, A.C.; Bowen, J.E.; Corti, D.; Veesler, D. Structure-guided covalent stabilization of coronavirus spike protein trimers in the closed conformation. *Nat. Struct. Biol.* **2020**, *27*, 942–949. [CrossRef] [PubMed]
13. Ray, D.; Le, L.; Andricioaei, I. Distant residues modulate conformational opening in SARS-CoV-2 spike protein. *Proc. Natl. Acad. Sci. USA* **2021**, *118*, e2100943118. [CrossRef]
14. Tegally, H.; Wilkinson, E.; Giovanetti, M.; Iranzadeh, A.; Fonseca, V.; Giandhari, J.; Doolabh, D.; Pillay, S.; San, E.J.; Msomi, N.; et al. Emergence and rapid spread of a new severe acute respiratory syndrome-related coronavirus 2 (SARS-CoV-2) lineage with multiple spike mutations in South Africa. *medRxiv* **2020**. [CrossRef]
15. Wang, P.; Casner, R.; Nair, M.; Huang, Y.; Shapiro, L.; Ho, D. Increased resistance of SARS-CoV-2 variant P.1 to antibody neutralization. *Cell Host Microbe* **2021**, *29*, 747–751. [CrossRef]
16. Wang, R.; Hozumi, Y.; Yin, C.; Wei, G.W. Decoding SARS-CoV-2 transmission and evolution and ramifications for COVID-19 diagnosis, vaccine, and medicine. *J. Chem. Inf. Model.* **2020**, *60*, 5853–5865. [CrossRef]
